# Novel N-Doped Carbon Dots/β-Cyclodextrin Nanocomposites for Enantioselective Recognition of Tryptophan Enantiomers

**DOI:** 10.3390/s16111874

**Published:** 2016-11-09

**Authors:** Qi Xiao, Shuangyan Lu, Chusheng Huang, Wei Su, Shan Huang

**Affiliations:** College of Chemistry and Materials Science, Guangxi Teachers Education University, Nanning 530001, China; qi.xiao@whu.edu.cn (Q.X.); shuangyan_lu@163.com (S.L.); huangcs@gxtc.edu.cn (C.H.); suwmail@163.com (W.S.)

**Keywords:** N-doped carbon dots, β-cyclodextrin, tryptophan enantiomers, enantioselective recognition, differential pulse voltammetry

## Abstract

Based on N-doped carbon dots/β-cyclodextrin nanocomposites modified glassy carbon electrodes (N-CDs/β-CD/GCE), an effective electrochemical sensor for enantioselective recognition of tryptophan (Trp) enantiomers was developed by differential pulse voltammograms (DPVs). Fluorescent N-CDs were synthesized through a hydrothermal method and characterized by spectroscopic approaches. The N-CDs/β-CD nanocomposites were efficiently electrodeposited on the surface of GCE through C–N bond formation between N-CDs and electrode. The obtained N-CDs/β-CD/GCE was characterized by multispectroscopic and electrochemical methods. Such N-CDs/β-CD/GCE generated a significantly lower Ip and more negative Ep in the presence of l-Trp in DPVs, which was used for the enantioselective recognition of Trp enantiomers. The N-CDs/β-CD nanocomposites showed different binding constants for tryptophan enantiomers, and they further selectively bonded with l-Trp to form inclusion complexes. This N-CDs/β-CD/GCE combined advantages of N-CDs with strong C–N binding ability and β-CD with specific recognition of Trp enantiomers to fabricate a novel sensing platform for enantioselective recognition of Trp enantiomers. This strategy provided the possibility of using a nanostructured sensor to discriminate the chiral molecules in bio-electroanalytical applications.

## 1. Introduction

The enantiomeric forms of chiral drugs usually show different pharmacological/metabolic behaviors in biological systems, and the demand for single-enantiomer drugs has substantially increased throughout the world, so the enantioselective recognition of enantiomeric forms of chiral molecules has attracted huge attention in the field of analytical and pharmaceutical research [1,2,3,4]. Since the natural amino acids in human beings are predominantly l-isomers and the presence of d-isomers in life forms usually indicates negative symptoms, the discrimination of amino acid enantiomers is thus of great interest and increasing importance in chemical, biological and pharmaceutical sciences [5,6]. Several analytical techniques have been explored for the enantiomers’ separation, such as high-performance liquid chromatography [6], capillary electrophoresis [7,8], and fluorescence spectrometry [9]. However, these methods require expensive chiral columns, relatively long analysis times and complicated sample pre-treatments. Recently, electrochemical approaches based on various chemically modified electrodes have been widely used in chiral recognition of propranolol [4], tyrosine [10], quinine and quinidine [11] due to their speed, simplicity, low cost, high sensitivity and enantioselective electrochemical signals.

l-Tryptophan (l-Trp), which plays a predominant role in all life forms, is a vital constituent of most proteins and precursor of serotonin, niacin and auxins [6,9]. On the other side, as a non-protein amino acid, d-Tryptophan (d-Trp) often presents different activity, toxicity and metabolic routes [12]. Therefore, it is necessary to distinguish between Trp enantiomers in the field of pharmaceuticals and biotechnology. Up to now, various electrochemical techniques have been developed for enantioselective determination of Trp enantiomers by building chiral surfaces for identifying the differences between Trp enantiomers. Among different types of chiral receptors, β-cyclodextrin (β-CD) has drawn great attention in enantioselective recognition and has emerged as an ideal host material to accommodate various amino acids enantiomers, due to its excellent chiral recognition and relatively low cost [13,14]. It has been reported that the hydrophobic indole ring of Trp isomers can penetrate into the cavity of β-CD to conform β-CD-Trp complex [15]. Due to the favorability of intermolecular hydrogen bonding formation between secondary hydroxyl groups of β-CD and amino groups of l-Trp compared to those of d-Trp, β-CD exhibits a higher affinity for l-Trp than d-Trp [15]. Recently, Kong’s group have reported the electrochemical recognition of Trp isomers by using β-CD or copper-modified β-CD [15,16,17], proving the possibility of β-CD-based electrochemical enantioselective recognition.

Fluorescent carbon dots (CDs) have attracted considerable attention in biological areas due to their high photostability, excellent photoluminescence and good biocompatibility [18]. In particular, N-doped CDs (N-CDs) draw great attention among researchers since nitrogen doping can effectively modulate the surface defects and greatly enhance the photoelectron property of CDs [19]. However, there are almost no relative reports on N-CDs-based enantioselective recognition of Trp enantiomers. Recently, Mo and co-workers reported that the combination of reduced graphene oxide and 1,10-phenanthroline copper(II) can achieve recognition of Trp enantiomers [20]. Yu et al. prepared a multi-walled carbon nanotube–chitosan composite for the chiral recognition of Trp enantiomers [21]. Kong’s group verified the possibility of graphene quantum dots-based Trp enantiomers recognition [17,22]. Since reduced graphene oxide, multi-walled carbon nanotube and graphene quantum dots all belong to the family of carbon nanomaterials, these reports suggest the feasibility of N-CDs-based electrochemical enantioselective recognition of Trp enantiomers.

**Scheme 1:**
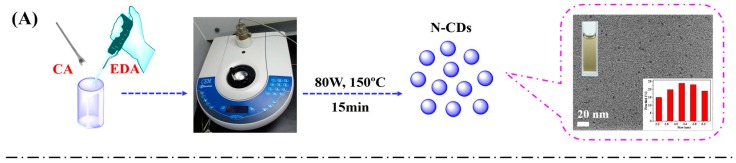

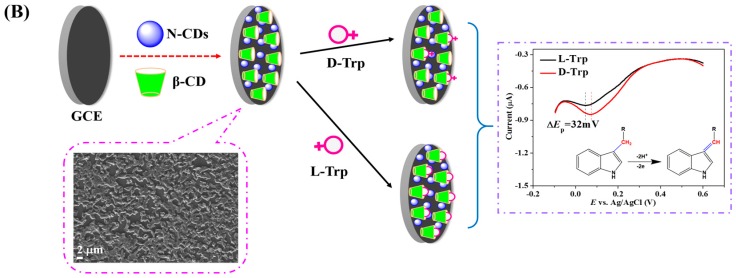
(**A**) Synthesis procedure of N-CDs by hydrothermal method. Inserts are HR-TEM image and diameter size distribution curve of N-CDs; (**B**) Fabrication process of N-CDs/β-CD/GCE and enantioselective recognition of Trp enantiomers. Inserts are SEM image of N-CDs/β-CD/GCE and DPVs responses of N-CDs/β-CD/GCE for l-Trp and d-Trp.

Inspired by these facts, we first report a novel electrochemical nanocomposite based on N-CDs and β-CD (N-CDs/β-CD) for the enantioselective recognition of Trp enantiomers. The synthesis procedure of N-CDs and stepwise fabrication process of N-CDs/β-CD/GCE are illustrated schematically in Scheme 1. Homogeneous N-CDs are synthesized through a hydrothermal method by using citric acid (CA) and ethylenediamine (EDA) as precursors. N-CDs/β-CD nanocomposites, which are synthesized by a self-assembly process through non-covalent interactions, can be effectively electrodeposited onto the surface of glassy carbon electrodes (GCE) due to the C–N bond formation between N-CDs and electrodes. The N-CDs/β-CD/GCE exhibited good capacitive performance and were capable of recognizing Trp enantiomers due to the hydrophobic central cavity of β-CD. A minor difference between Trp enantiomers and nanocomposites was identified directly, and an obvious difference of oxidation potential signals for d-Trp and l-Trp was observed in differential pulse voltammograms (DPVs). These N-CDs/β-CD nanocomposites may provide an alternative strategy for the design of novel electrochemical enantioselective sensors with superior performance.

## 2. Materials and Methods

### 2.1. Reagents

l-Trp (99%) and d-Trp (99%) were purchased from Aladdin Inc. (Shanghai, China). CA, EDA and β-CD were obtained from Sinopharm Chemical Reagent Factory (Shanghai, China). Tris, HCl, NaH_2_PO_4_, Na_2_HPO_4_, NaCl, KCl, CH_3_COOH, CH_3_COONa, K_2_Fe(CN)_6_, K_3_Fe(CN)_6_, H_2_SO_4_ and ethanol were of analytical reagent grade and used as received without any further purification. Riboflavin sample was purchased from Guangji Pharmaceutical Co., Ltd. (Wuxue, China). Firstly, 50 mg of riboflavin table was mixed with 2 mL PB buffer (pH 7.0). After 20 min sonication, the mixture was centrifuged for 20 min, and then supernatant was filtrated with 0.22 μm membrane. The filtrate was collected for further application. Ultrapure water with resistivity of 18.2 MΩ·cm was produced by passing through RiOs 8 unit followed by Millipore-Q Academic purification set (Millipore, Bedford, MA, USA).

### 2.2. Apparatus

All electrochemical experiments were performed on CHI-760E electrochemical workstation (Shanghai Chenhua Instrument Co., Ltd., Shanghai, China). A three-electrode cell was employed, and GCE or modified GCE was used as working electrode. Ag/AgCl electrode served as the reference electrode and platinum wire was employed as the counter electrode. N-CDs were synthesized by using CEM Discover Benchmate microwave reactor (CEM, Matthews, NC, USA). UV-vis absorption spectra were recorded on Cary 100 UV-vis spectrophotometer (Agilent Technologies, Inc., Agilent, Australia). Fluorescence spectra were recorded on RF-5301 PC luminescence spectrometer (Shimadzu Co., Ltd., Tokyo, Japan). Time-resolved fluorescence decay traces were recorded with Fluorolog-3 system (Horiba JobinYvon, Paris, France). FT-IR spectra were recorded on Nicolet iS10 spectrometer (Thermo, Waltham, MA, USA). Circular dichroism (CD) spectra were recorded on Chirascan CD spectrometer (Applied Photophysics, Surrey, UK). Scanning electron microscopy (SEM) images were taken on MERLIN Compact field emission scanning electron microscope (ZEISS, Jena, Germany). High-resolution transmission microscopy (HR-TEM) images were recorded on JEM-2100 transmission electron microscope (JEOL, Tokyo, Japan). X-ray photoelectron spectroscopic (XPS) studies were performed by ESCALAB 250 spectrometer (Thermo Fisher Scientific, Waltham, MA, USA). All pH measurements were made with a basic pH meter PB-10 (Sartorius Scientific Instruments Co., Ltd., Beijing, China).

### 2.3. Preparation of N-CDs

N-CDs were synthesized by a hydrothermal method according to the literature after minor modifications [23]. Briefly, 0.4203 g CA and 0.4 mL EDA were mixed and dissolved into 5.0 mL ultrapure water. The mixture was placed in 80 W microwave oven at 150 °C for 15 min. After the reaction, the reactors were cooled to room temperature naturally and then a dark-yellow N-CDs solution was obtained (Scheme 1A). The product was subjected to dialysis in order to obtain N-CDs. Finally, the obtained N-CDs were stored at 4 °C for application.

### 2.4. Preparation of N-CDs/β-CD/GCE

Before modification, bare GCE were polished with 1.0 μm, 0.3 μm and 0.05 μm alumina slurry on a polishing cloth, rinsed with ultrapure water thoroughly and then sonicated in ethanol, 0.5 M H_2_SO_4_ and ultrapure water for 1 min, respectively. For N-CDs/β-CD/GCE preparation, 113.5 mg β-CD was added into 10 mL N-CDs solution (2 mg·mL^−1^) to obtain N-CDs/β-CD nanocomposites. Finally, homogenous N-CDs/β-CD nanocomposites were electrodeposited on the surface of pretreated GCE by cyclic voltammetry (CV) in the potential range of 0–1.0 V with the scan rate of 100 mV·s^−1^ for 50 cycles.

### 2.5. Enantioselective Recognition of Trp Enantiomers

CV measurements were carried out in 5.0 mM [Fe(CN)_6_]^3−^/^4−^ solution containing 0.1 M KCl between 0.00 and 0.70 V at a scan rate of 50 mV·s^−1^. Electrochemical impedance spectrometry (EIS) experiments were performed in 5.0 mM [Fe(CN)_6_]^3−^/^4−^ solution containing 0.1 M KCl over the frequency range from 10^5^ Hz to 0.1 Hz. The amplitude was 0.005 V. Electrochemical responses on N-CDs/β-CD/GCE to l-Trp or d-Trp in 10 mM PB (pH 7.0) were carefully investigated by DPVs with instrumental parameters as: potentials range from −0.1 to 0.6 V; pulse amplitude 0.05 V; pulse width 0.05 s; sampling width 0.0167 s; pulse period 0.5 s; quiet time 2 s; scan rate 0.008 V·s^−1^. The recognition efficiency was evaluated by monitoring the differential oxidation peak potential in DPVs. Due to the preferential inclusion complexation between β-CD and l-Trp, the N-CDs/β-CD nanocomposites can be used to separate Trp enantiomers.

## 3. Results and Discussion

### 3.1. Characterization of N-CDs and N-CDs/β-CD Nanocomposites

**Figure 1:**
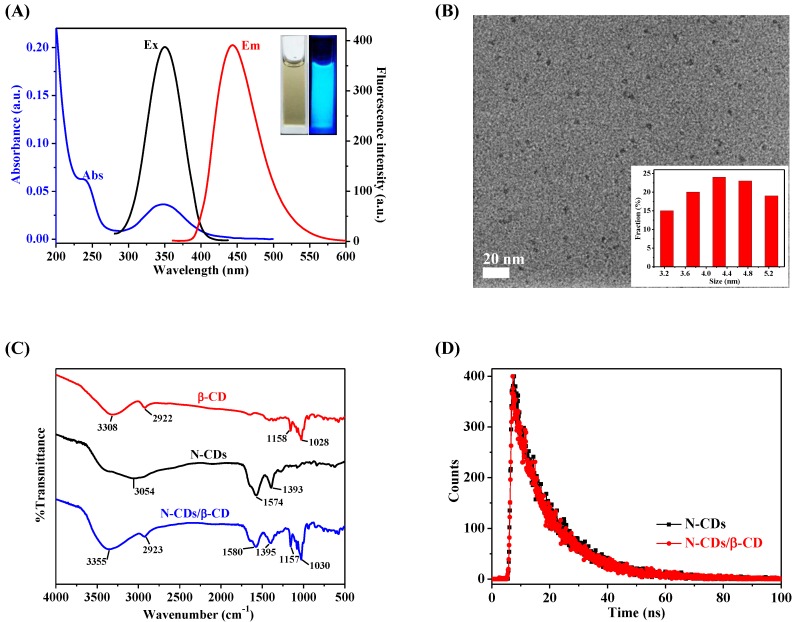
(**A**) UV-vis absorption spectra of N-CDs, excitation spectrum of N-CDs at *λ*_em_ = 443 nm and emission spectrum of N-CDs at *λ*_ex_ = 350 nm. Insert: Photograph of N-CDs under the radiation of white light (Left) and 365 nm UV lamp (Right); (**B**) HR-TEM image of N-CDs. Insert: Diameter size distribution curve of N-CDs; (**C**) FT-IR spectrum of N-CDs, β-CD and N-CDs/β-CD nanocomposites; (**D**) Fluorescence decay traces of N-CDs without and with β-CD at *λ*_ex/em_ = 350 nm/443 nm.

Figure 1A displays UV-vis absorption and fluorescence spectra of N-CDs. It is clear that N-CDs exhibit broad absorption bands from 200 nm to 500 nm with an obvious UV-vis absorption peak centered at 282 nm. These N-CDs show an obvious, narrow and almost symmetrical fluorescence spectrum with peak position of 443 nm under excitation wavelength of 350 nm, which is the same as the reported N-CDs nanomaterials [23]. From the digital pictures inserted in Figure 1A, the diluted N-CDs solution is tawny under ambient daylight but exhibits strong blue emission under UV light (365 nm), which confirms the good fluorescent property of N-CDs. The solution stability of N-CDs was further investigated by monitoring of fluorescence intensity in a wide pH range from 4.0 to 11.0. The results point out that the fluorescence intensity of N-CDs at 443 nm changes slightly, suggesting that these N-CDs possess high stability in a wide pH range (Figure S1). Moreover, a HR-TEM image indicates that these N-CDs are nearly spherical with good size distribution and excellent monodispersity (Figure 1B). The average diameter of N-CDs is estimated to be around 4.3 nm (insert in Figure 1B).

N-CDs/β-CD nanocomposites were firstly identified by FT-IR spectrometry. As shown in Figure 1C, for pure N-CDs, the absorption peak at 1393 cm^−1^ is assigned to C–H bending vibrations, and the absorption peaks at 3054 cm^−1^ and 1574 cm^−1^ are attributed to the stretching vibrations and bending vibrations of N–H bond, respectively [24]. For pure β-CD, the characteristic peaks at 1028 cm^−1^ and 1158 cm^−1^ are attributed to the stretching vibration of C–O–C bond, and two peaks at 2922 cm^−1^ and 3308 cm^−1^ are assigned to the stretching vibrations of C–H and O–H bonds, respectively [17]. All characteristic peaks of N-CDs and β-CD are still observed in the spectra of N-CDs/β-CD nanocomposites, suggesting the successful attachment of β-CD to N-CDs. It is noteworthy that the stretching vibrations of N–H bond of N-CDs (1574 cm^−1^) and O–H bond of β-CD (3308 cm^−1^) are shifted to 1580 cm^−1^ and 3355 cm^−1^ at N-CDs/β-CD nanocomposites, respectively, which is ascribed to the hydrogen bonding between the amino groups of N-CDs and the hydroxyl groups of β-CD [17]. Moreover, N-CDs/β-CD nanocomposites exhibit broader absorption in high wavenumber range from 3000 cm^−1^ and 3750 cm^−1^, reconfirming the formation of hydrogen bonds between N-CDs and β-CD.

N-CDs/β-CD nanocomposites were then characterized through time-resolved fluorescence spectrometry by monitoring the variation of fluorescence lifetime of N-CDs. The fluorescence decay curves of N-CDs and N-CDs/β-CD nanocomposites are shown in Figure 1D. It is hard to judge the fluorescence lifetimes of N-CDs and N-CDs/β-CD nanocomposites, since two fluorescence decay curves are almost overlapped. However, the fluorescence decay curves of N-CDs are fitted well with mono-exponential equation, so N-CDs show one fluorescence decay component [25]. As exhibited in Figure 1D, the fluorescence lifetimes of N-CDs and N-CDs/β-CD nanocomposites are about 13.61 ns and 13.36 ns, respectively. The fluorescence lifetime of N-CDs changes slightly in the presence of β-CD within experimental errors, which further proves the complex formation between N-CDs and β-CD. These results indicate that N-CDs bond with β-CD to form novel N-CDs/β-CD complex mainly through hydrogen bonding forces.

**Figure 2:**
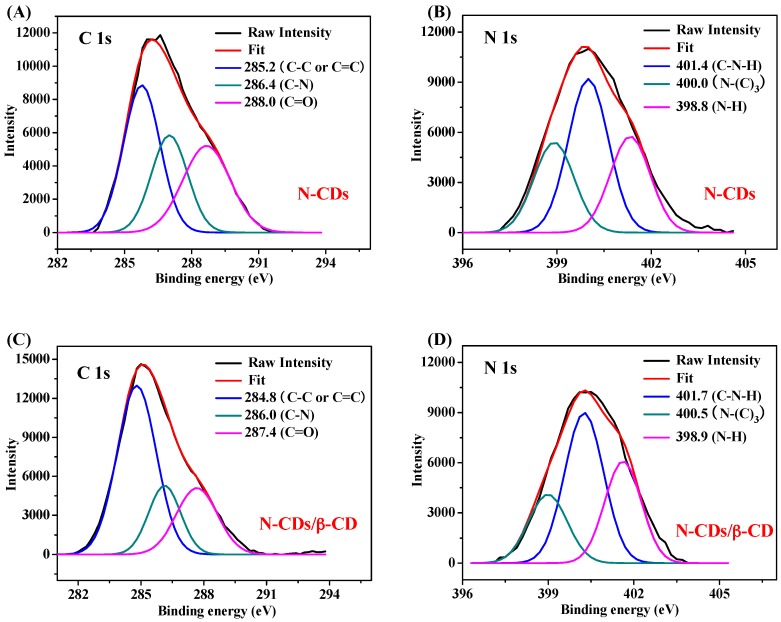
High-resolution C 1s (**A**) and N 1s (**B**) XPS spectra of N-CDs; High-resolution C 1s (**C**) and N 1s (**D**) XPS spectra of N-CDs/β-CD nanocomposites.

N-CDs/β-CD nanocomposites were continuously characterized and evidenced by XPS. As shown in Figure 2A, three typical characteristic peaks of N-CDs at 285.2 eV (40.9%), 286.4 eV (27.2%) and 288.0 eV (31.8%) are observed in high-resolution C 1s spectrum. The peaks at 285.2 eV, 286.4 eV and 288.0 eV correspond to the sp3 C in C–C bond or sp2 C in C=C bond, sp3 C in C–N bond, and sp2 C in C=O bond, respectively [26], suggesting three unit moieties of C 1s: graphitic or aliphatic (C=C and C–C), nitrous, and oxygenated. The high-resolution N 1s spectrum of N-CDs also exhibits three peaks at 398.8 eV (26.5%), 400.0 eV (45.2%) and 401.4 eV (28.3%) (Figure 2B), which are associated with N–H, N–(C)3 and C–N–H bonds, respectively [26]. These three peaks reveal two different types of nitrogen doping: pyrrolic nitrogen and graphitic nitrogen, which is highly consistent with the reported results [27]. As shown in Figure 2C,D, N-CDs/β-CD nanocomposites also show three peaks in high-resolution C 1s spectrum at 284.8 eV (57.3%), 286.0 eV (19.1%) and 287.4 eV (23.6%) and in high-resolution N 1s spectrum at 398.9 eV (21.8%), 400.5 eV (47.8%) and 401.7 eV (30.4%), respectively. The elemental analysis of the surface of C in N-CDs and N-CDs/β-CD gives 60.8% and 62.0%, respectively. The elemental analysis of the surface of N in N-CDs and N-CDs/β-CD are 15.3% and 13.1%, respectively. The high content of C is due to the presence of β-CD, while the contribution at low content of N is due to the less presence of N-containing groups. XPS data demonstrate that the main chemical bond in N-CDs/β-CD nanocomposites is hydrogen bond, which is agreed with the FT-IR reports.

### 3.2. Electrochemical Characteristics of N-CDs/β-CD/GCE

**Figure 3:**
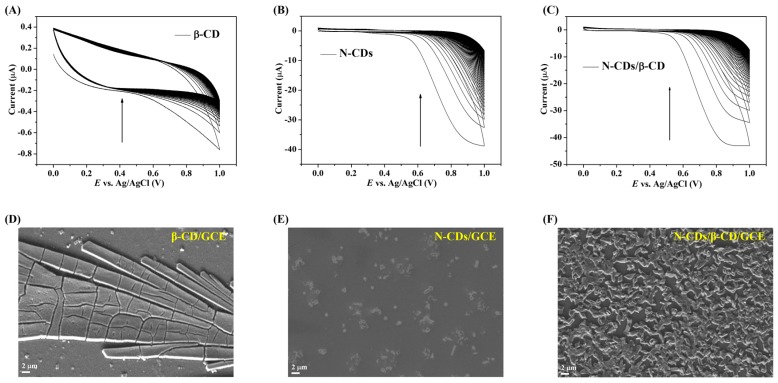
CVs of β-CD (**A**); N-CDs (**B**) and N-CDs/β-CD nanocomposites (**C**) at GCE surface; Scan rate: 100 mV·s^−1^; scan potential: 0 to 1.0 V; consecutive cycles: 50; SEM images of β-CD/GCE (**D**); N-CDs/GCE (**E**) and N-CDs/β-CD/GCE (**F**).

Homogenous N-CDs/β-CD nanocomposites were electrodeposited on GCE surface by CV in the potential range of 0–1.0 V with the scan rate of 100 mV·s^−1^ for 50 cycles. As shown in Figure 3A–C, due to the direct oxidation of pure β-CD, pure N-CDs and N-CDs/β-CD nanocomposites at the electrode surface, well-defined oxidation peaks with large potential currents appear in modified GCE when potential sweeps from 0 to 1.0 V at the first scan. With the increment of scan cycles, the growth of different surface films becomes much slower and peak currents decrease correspondingly. The oxidation peaks finally disappear after the complete formation of three different films at GCE surface, which is highly consistent with the reported literature [28].

N-CDs/β-CD/GCE was further verified by a microscopy technique. The SEM images of β-CD/GCE, N-CDs/GCE and N-CDs/β-CD/GCE are shown in Figure 3D–F. As can be seen, a random rod-like β-CD is formed on the surface of GCE (Figure 3D), and an irregular distribution of N-CDs on GCE surface is also observed (Figure 3E). However, the majority of GCE surface is still uncovered by pure β-CD and pure N-CDs, ascribed to the insulating feature and weak binding ability of β-CD and the good water-solubility of N-CDs. This situation is quite different for N-CDs/β-CD nanocomposites modified GCE, because a compact and homogeneous N-CDs/β-CD film is formed on the GCE surface (Figure 3F). Many kinds of graphene nanomaterials are reported to be used for surface modification of GCE through a strong π–π stacking interaction [29] while amino functionalized carbon-based nanomaterials can be fixed on the electrode surface usually through C–N bond formation. The formation of N-CDs/β-CD nanocomposites cannot only maintain the strong binding ability of N-CDs on GCE surface but also efficiently enhance the hydrophobic property of N-CDs and β-CD. Therefore, when potential sweeps from 0 to 1.0 V are exerted on GCE, the electrodeposited amount of N-CDs/β-CD nanocomposites on the GCE surface increases significantly. Finally, a compact and homogeneous N-CDs/β-CD film is established on the GCE surface.

**Figure 4:**
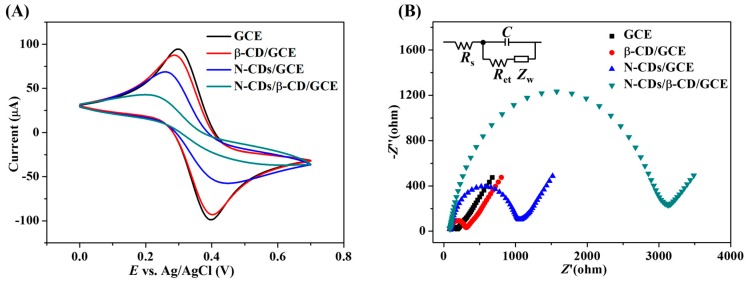
(**A**) CVs of bare GCE, β-CD/GCE, N-CDs/GCE and N-CDs/β-CD/GCE in 5.0 mM[Fe(CN)_6_]^3−^/^4−^ solution containing 0.1 M KCl; (**B**) Nyquist plots of bare GCE, β-CD/GCE, N-CDs/GCE and N-CDs/β-CD/GCE in 5.0 mM [Fe(CN)_6_]^3−^/^4−^ solution containing 0.1 M KCl with the frequencies swept from 10^5^ Hz to 0.1 Hz. Insert is the equivalent circuit for fitting the plots. *R*_s_: solution resistance; *R*_et_: electron transfer resistance; *C*: interfacial double layer capacitance; *Z*_W_: Warburg impedance.

Electrochemical characterizations of N-CDs/β-CD nanocomposites also indicate the successful combination of N-CDs and β-CD. Figure 4A shows the CVs of bare GCE, β-CD/GCE, N-CDs/GCE and N-CDs/β-CD/GCE in 5.0 mM [Fe(CN)_6_]^3−^/^4−^ solution containing 0.1 M KCl. As can be seen, a pair of well-defined reversible redox peaks belonging to redox couple [Fe(CN)_6_]^3−^/^4−^ exhibits at bare GCE. After the electrodeposition of β-CD on the surface of GCE, two peak currents decrease slightly due to the inferior conductivity of β-CD [17]. Electrodeposition of N-CDs on GCE surface dramatically decreases two peak currents and obviously increases the peak potential difference, because N-CDs can efficiently hinder the electron transfer and partially block the diffusion of [Fe(CN)_6_]^3−^/^4−^ to electrode surface [30]. Due to the synergistic effect of N-CDs and β-CD, two peak currents are further decreased at the N-CDs/β-CD nanocomposites deposited GCE. These results not only confirm the successful deposition of three substances but also prove the non-covalent combination of N-CDs and β-CD.

EIS of bare GCE and different substances modified GCE were continuously investigated. Figure 4B shows the typical Nyquist plots for bare GCE, β-CD/GCE, N-CDs/GCE and N-CDs/β-CD/GCE in 5.0 mM [Fe(CN)_6_]^3−^/^4−^ solution containing 0.1 M KCl with the frequencies swept from 10^5^ Hz to 0.1 Hz. The data are fitted well with the equivalent circuit based on Z-view software (inserted in Figure 4B). In the inserted circuit model, *C* is the interfacial double layer capacitance, *R*_s_ is the solution resistance, *R*_et_ is the electron transfer resistance and *Z*_w_ is the Warburg impedance, respectively. It is obvious that EIS exhibit the same change tendency as CVs, the calculated *R*_et_ values of bare GCE, β-CD/GCE, N-CDs/GCE and N-CDs/β-CD/GCE are 88.7 Ω, 225.2 Ω, 963.5 Ω and 2993.3 Ω, respectively. Bare GCE displays the lowest *R*_et_ value and N-CDs/β-CD/GCE exhibits the highest *R*_et_ value, suggesting that the resistance at the interface of electrode-solution is much more significant at N-CDs/β-CD/GCE than those at β-CD/GCE and N-CDs/GCE. On a side-note, the presence of N-CDs/β-CD nanocomposites significantly hinders the electron transfer between the redox couple [Fe(CN)_6_]^3−^/^4−^ and the electrode interface.

**Figure 5:**
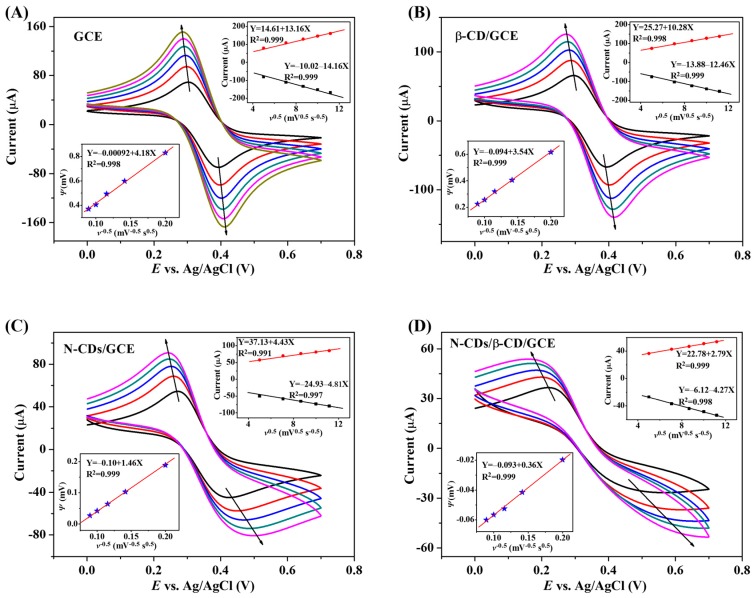
CVs and associated kinetic analyses at bare GCE (**A**); β-CD/GCE (**B**); N-CDs/GCE (**C**) and N-CDs/β-CD/GCE (**D**) in 5.0 mM [Fe(CN)_6_]^3−^/^4−^ solution containing 0.1 M KCl. The inserts are the corresponding plots of *ψ*−*v*^−0.5^ curves and *I*−*v*^0.5^ curves. Sweep rates: 25, 50, 75, 100 and 125 mV·s^−1^.

The influences of scan rates on the electrochemical response of bare GCE and different substances modified GCE were studied. As shown in Figure 5, all electrodes show the reversible characteristics at scan rates from 25 mV·s^−1^ to 125 mV·s^−1^. In particular, with the increase of scan rate, two peak currents of all electrodes increase gradually. Furthermore, both oxidation peak currents and reduction peak currents are directly proportional to the square root of scan rate (*v*) over the range from 25 mV·s^−1^ to 125 mV·s^−1^ (insert in Figure 5), suggesting that the electrochemical processes of [Fe(CN)_6_]^3−^/^4−^ at the surface of four electrodes are controlled by the diffusion process [30,31]. Moreover, the standard electrochemical rate constant (*k*^Θ^) of different electrodes can be calculated according to the following equations [32]:

(1) $$\psi =k^{\Theta }{\left(\frac{D_{O}}{D_{R}}\right)}^{\alpha /2}\sqrt{\frac{RT}{\pi nFD_{O}v}}$$

(2) $$\psi =\frac{-0.6288+0.0021n\Delta E_{P}}{1-0.017n\Delta E_{P}}$$

Herein, *ψ* is the dimensionless kinetic parameter, Δ*E*_p_ is the peak potential separation, *v* is the sweep rate, *α* is the charge transfer coefficient, and *D*_O_ and *D*_R_ are the diffusion coefficients of [Fe(CN)_6_]^3−^/^4−^, respectively. *F*, *R* and *T* have their usual meanings. In most cases, the value of *α* is 0.5, and the values of *D*_O_ and *D*_R_ are 7.63 × 10^−6^ cm^2^·s^−1^ and 6.32 × 10^−6^ cm^2^·s^−1^, respectively [29,33]. According to the above equations, the value of *k*^Θ^ can be calculated by the dependence of *ψ* on the inverse square root of sweep rate *v*^−0.5^. The calculated average values of *k*^Θ^ for bare GCE, β-CD/GCE, N-CDs/GCE and N-CDs/β-CD/GCE are 3.85 × 10^−3^ cm·s^−1^, 3.27 × 10^−3^ cm·s^−1^, 1.35 × 10^−3^ cm·s^−1^ and 3.41 × 10^−4^ cm·s^−1^, respectively. The *k*^Θ^ value of N-CDs/β-CD/GCE is therefore much lower than those of other substances modified GCE, indicating the slower electron transfer rate of N-CDs/β-CD/GCE.

### 3.3. Binding Interactions of N-CDs/β-CD Nanocomposites with Trp

**Figure 6:**
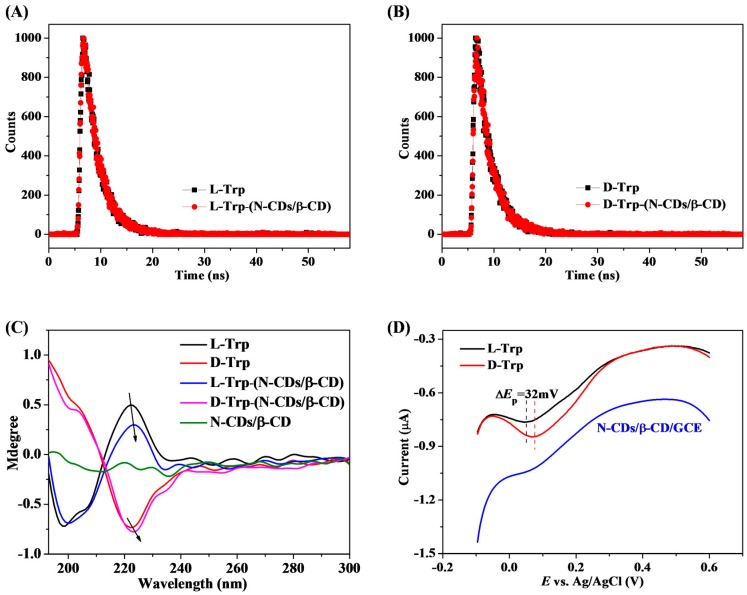
Fluorescence decay traces of l-Trp (**A**) and d-Trp (**B**) without and with N-CDs/β-CD nanocomposites at *λ*_ex/em_ = 278 nm/350 nm; (**C**) CD spectra of l-Trp, d-Trp, N-CDs/β-CD nanocomposites, N-CDs/β-CD–l-Trp and N-CDs/β-CD–d-Trp; (**D**) DPVs responses of N-CDs/β-CD/GCE for l-Trp and d-Trp.

Interactions between N-CDs/β-CD nanocomposites and Trp enantiomers were firstly investigated by time-resolved fluorescence spectrometry through monitoring the fluorescence lifetimes of Trp enantiomers. The fluorescence decay curves of l-Trp and d-Trp before and after the addition of N-CDs/β-CD nanocomposites are shown in Figure 6A,B. All results are fitted with mono-exponential equations [25]. As inserted in Figure 6A,B, the fluorescence lifetimes of l-Trp and d-Trp are all 2.86 ns. After the addition of N-CDs/β-CD nanocomposites, the fluorescence lifetimes of l-Trp and d-Trp change slightly to 2.72 ns and 2.76 ns within experimental errors. Since Trp enantiomers can enter into the hydrophobic central cavity of β-CD to form an inclusion complex, the complexes’ formation between N-CDs/β-CD nanocomposites and Trp enantiomers is further validated.

Binding interactions between N-CDs/β-CD nanocomposites and Trp enantiomers were then investigated by CD spectrometry [34,35]. Figure 6C represents the differential CD spectra of l-Trp and d-Trp interacting with N-CDs/β-CD nanocomposites. The strong absorption peaks of Trp enantiomers near 220 nm are attributed to the *n*−π* transition that occurs mainly due to NH–C=O stretching vibration and benzene ring of Trp enantiomers [6]. On the other hand, N-CDs/β-CD nanocomposites exhibit almost no absorption in the same wavelength range. Furthermore, after the addition of N-CDs/β-CD nanocomposites, the absorption peaks of both l-Trp and d-Trp exhibit a tiny red-shift from 222 nm to 223 nm, implying the binding interactions between N-CDs/β-CD nanocomposites and Trp enantiomers. Meanwhile, the ellipticity (mdegree) value of l-Trp decreases but that of d-Trp increases. The variation in ellipticity value is much bigger for l-Trp; therefore, l-Trp is more prone to binding with N-CDs/β-CD nanocomposites and forming stable N-CDs/β-CD–l-Trp inclusion complex (Scheme 1).

Binding interactions between N-CDs/β-CD nanocomposites and Trp enantiomers were finally researched by DPVs. As illustrated in Figure 6D, the DPVs obtained at N-CDs/β-CD/GCE shows unobvious oxidation peak, which is regarded as the blank response. As can be seen, after the addition of Trp enantiomers, the peak current of N-CDs/β-CD/GCE decreases, ascribed to the inclusion complexes’ formation. In addition, the inclusion complex of N-CDs/β-CD–l-Trp generates a significantly lower peak current than N-CDs/β-CD–d-Trp, and the peak current ratio of N-CDs/β-CD–d-Trp to N-CDs/β-CD–l-Trp (*I*_D_/*I*_L_) is calculated to be 1.11 (i.e., −0.8467 μA to −0.7644 μA), which indicates that N-CDs/β-CD preferably includes with l-Trp compared with d-Trp due to the favorability of hydrogen bond formation between β-CD and l-Trp [15,17]. Moreover, the differential oxidation peak potential (∆*E*_p_) between N-CDs/β-CD–d-Trp and N-CDs/β-CD–l-Trp is 32 mV, which can be used for the enantioselective recognition of Trp enantiomers. Pre-experimental results indicate that although the tapered hydrophobic cavities of β-CD are capable of including Trp enantiomers [15], the enantiorecognition efficiency at β-CD/GCE is dramatically inferior to that of N-CDs/β-CD/GCE (∆*E*_p_ = 8 mV; Figure S2). Due to the insulating feature and weak adsorption ability of β-CD, small amount of β-CD is electrodeposited on GCE surface, which results in the deteriorated recognition efficiency of β-CD/GCE. These phenomena indicate that the constructed N-CDs/β-CD/GCE is an excellent electrochemical sensor for enantioselective recognition of Trp enantiomers.

### 3.4. Electrocatalytic Properties of N-CDs/β-CD/GCE for Trp Enantiomers

#### 3.4.1. Influence of Supporting Electrolytes

**Figure 7:**
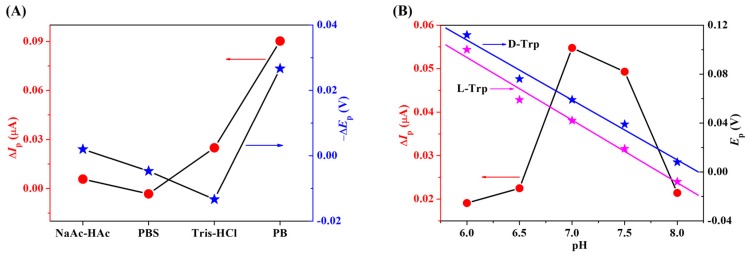
Influences of supporting electrolytes (**A**) and pH (**B**) on enantiorecognition efficiency of N-CDs/β-CD/GCE toward Trp enantiomers. Errors bars represent the standard deviation for three independent measurements.

Influence of supporting electrolytes on N-CDs/β-CD/GCE was investigated by DPVs in 10 mM of NaAc-HAc, PBS (KH_2_PO_4_-Na_2_HPO_4_-NaCl-KCl), Tris-HCl and PB (NaH_2_PO_4_-Na_2_HPO_4_) buffer solutions with pH 7.0. Figure 7A shows the oxidation peak current difference (∆*I*_p_) and the oxidation peak potential difference (∆*E*_p_) between N-CDs/β-CD–l-Trp and N-CDs/β-CD–d-Trp in four different buffer solutions. It is obvious that the values of both ∆*I*_p_ and ∆*E*_p_ in PB solution are much bigger than those in other buffer solutions, suggesting that the inclusion complex formation of N-CDs/β-CD–l-Trp is promoted in PB solution compared with other buffer solutions. It has been reported that some supporting electrolytes, especially PB buffer solution, can effectively reduce the repulsion of biomolecules in solutions [36], so the inclusion complexes’ formation between N-CDs/β-CD nanocomposites and Trp enantiomers easily took place in PB solution. Therefore, 10 mM PB buffer solution is selected as the suitable supporting electrolyte for the following measurements.

#### 3.4.2. pH Effect

Effect of pH for the inclusion complexes formation between N-CDs/β-CD nanocomposites and Trp enantiomers is also significant. Therefore, effect of pH in PB solution on the electrochemical behavior of Trp enantiomers at N-CDs/β-CD/GCE was investigated by DPVs. The plot of ∆*I*_p_ between l-Trp and d-Trp at N-CDs/β-CD/GCE against pH values is shown in Figure 7B. When pH value increases from 6.0 to 8.0, ∆*I*_p_ value increases gradually from pH 6.0 to pH 7.0 and then decreases dramatically from pH 7.0 to pH 8.0. Since the increased acidity will destroy the natural polysaccharide structure of β-CD and the excessively high alkalinity can increase the water-solubility of N-CDs [17], both excessive acidic and alkaline environments are disadvantageous to the electrochemical enantiorecognition of Trp enantiomers by using N-CDs/β-CD nanocomposites. As can be seen from Figure 6B, the maximum ∆*I*_p_ value is observed at pH 7.0, so 10 mM PB solution with pH 7.0 is selected as the optimal buffer solution for electrochemical enantioselective recognition of Trp enantiomers.

In addition, the variation of peak potential (*E*_p_) against pH values is usually used to explain the electrochemical reaction mechanism involving protons and electrons. The plots of oxidation peak potentials of Trp enantiomers at N-CDs/β-CD/GCE against pH values are also exhibited in Figure 7B. When the pH value increases from 6.0 to 8.0, *E*_p_ values of N-CDs/β-CD–l-Trp and N-CDs/β-CD–d-Trp shift to the negative direction, suggesting the participation of protons in the oxidation process of Trp enantiomers on N-CDs/β-CD/GCE surface [37]. The good linear regression equations between *E*_p_ of and pH values are *E*_pa_(V) = −0.049 pH + 0.4018 with correlation coefficient of 0.9921 for N-CDs/β-CD–d-Trp and *E*_pa_(V) = −0.0512 pH + 0.4008 with correlation coefficient of 0.9904 for N-CDs/β-CD–l-Trp, respectively. The slopes of two regression equations are calculated as 0.049 V·pH^−1^ (N-CDs/β-CD–d-Trp) and 0.0512 V·pH^−1^ (N-CDs/β-CD–l-Trp), respectively. Two slopes are close to the theoretical value of 0.0592 V·pH^−1^ (25 °C), indicating the equal numbers of the participated electron and proton during the electrochemical oxidation process of Trp enantiomers on the surface of N-CDs/β-CD/GCE (Scheme 1B) [37,38].

#### 3.4.3. Binding Ratio and Binding Constant

Trp enantiomers can enter into the hydrophobic cavity of β-CD in N-CDs/β-CD nanocomposites to form inclusion complexes according to the following reaction schemes:

(3) $$\mathrm{N-CDs}/\beta \mathrm{-CD}+m\mathrm{Trp}=\mathrm{N-CDs}/\beta {\mathrm{-CD}\mathrm{-Trp}}_{m}$$

(4) $$K_{a}^{m}=[\mathrm{N-CDs}/\beta {\mathrm{-CD}\mathrm{-Trp}}_{m}]/([\mathrm{N-CDs}/\beta \mathrm{-CD}]{\left[\mathrm{Trp}\right]}^{m})$$

Herein, *m* is the binding number or Hill coefficient and *K*_a_ is the association equilibrium constant. According to the overall Hill cooperativity model, the fraction (*f*) of Trp enantiomers bond to N-CDs/β-CD is described by the following equations:

(5) $$\begin{array}{ll} f & =[\mathrm{N-CDs}/\beta \mathrm{-CD}{\mathrm{-Trp}}_{m}]/({\left[\mathrm{N-CDs}/\beta \mathrm{-CD}{\mathrm{-Trp}}_{m}\right]}_{\max})\\ & =[\mathrm{N-CDs}/\beta \mathrm{-CD}{\mathrm{-Trp}}_{m}]/([\mathrm{N-CDs}/\beta \mathrm{-CD}{\mathrm{-Trp}}_{m}]+[\mathrm{Trp}]) \end{array}$$

Herein, [N-CDs/β-CD-Trp_m_]_max_ is the maximum concentration of N-CDs/β-CD-Trp_m_ in solution, [N-CDs/β-CD-Trp_m_] is the concentration of N-CDs/β-CD-Trp_m_ and [Trp] is the concentration of uncombined Trp enantiomers, respectively. The complicated relationships among *m*, *K*_a_, *f* and the oxidation peak current difference of Trp (∆*I*_p_) can be expressed by the following equations:

(6) $$\log[f/(1-f)]=m\log(K_{a}/M)+m\log([\mathrm{Trp}]/M)$$

(7) $$\log[\Delta I_{p}/(\Delta I_{\mathrm{pmax}}-\Delta I_{p})]=m\log(K_{a}/M)+m\log([\mathrm{Trp}]/M)$$

**Figure 8:**
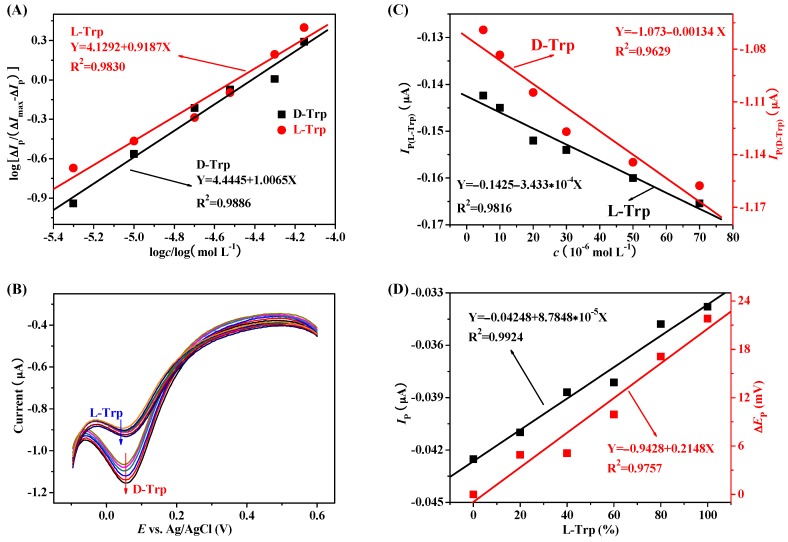
(**A**) Plots of log[∆*I*_p_/(∆*I*_pmax_−∆*I*_p_)] versus log([d-Trp]/M) and log([l-Trp]/M); (**B**) DPVs responses of N-CDs/β-CD/GCE for l-Trp and d-Trp. The concentrations of Trp enantiomers are 0, 5.0 × 10^−^^6^ M, 1.0 × 10^−^^5^ M, 2.0 × 10^−^^5^ M, 3.0 × 10^−^^5^ M, 5.0 × 10^−^^5^ M and 7.0× 10^−^^5^ M, respectively; (**C**) Plots of *I*_p_ versus the concentrations of d-Trp (Red line) and l-Trp (Black line); (**D**) Plots of *I*_p_ (Black line) and ∆*E*_p_ (Red line) versus the percentage of l-Trp.

According to the above equations, the value of *m* can be calculated from the plot of log[∆*I*_p_/(∆*I*_pmax_−∆*I*_p_)] against log([Trp]/M). As indicated in Figure 8A, good linear relationships are concluded as log[∆*I*_p_/(∆*I*_pmax_−∆*I*_p_)] = 4.4445 + 1.0065log([d-Trp]/M) with correlation coefficient of 0.9886 and log[∆*I*_p_/(∆*I*_pmax_−∆*I*_p_)] = 4.1292 + 0.9187log([l-Trp]/M) with a correlation coefficient of 0.9830, respectively. The binding numbers between N-CDs/β-CD and Trp enantiomers are calculated as around one. These results imply that Trp enantiomers can form single inclusion complex with N-CDs/β-CD, and the stoichiometry of the cooperative Trp enantiomers binding is thus one N-CDs/β-CD molecule, which are highly consistent with the reported results [15,17]. Moreover, the association equilibrium constants of N-CDs/β-CD–d-Trp and N-CDs/β-CD–l-Trp are 2.60 × 10^4^ M^−1^ and 3.12 × 10^4^ M^−1^, respectively, implying that binding ability of N-CDs/β-CD to l-Trp is much stronger, which is also agreed with the results obtained from DPVs’ experiments.

### 3.5. Electroanalytical Performance

N-CDs/β-CD nanocomposites modified GCE was firstly used to detect Trp enantiomers one by one through DPVs. The DPVs of different concentrations of l-Trp and d-Trp on N-CDs/β-CD/GCE are shown in Figure 8B. It is obvious that the DPV peak currents increase with the increment of the concentrations of l-Trp and d-Trp. As shown in Figure 8C, the DPV peak currents are linearly dependent on the concentrations of Trp enantiomers in the range of 5.0 × 10^−^^6^ M to 7.0× 10^−^^5^ M. As indicated in Figure 8C, two regression equations are concluded as *I*_p_ = −1.073 − 1340[d-Trp] with correlation coefficient of 0.9629 and *I*_p_ = −0.1425 − 343.3[l-Trp] with a correlation coefficient of 0.9816, respectively. The detection limits of d-Trp and l-Trp are 1.7 × 10^−^^6^ M and 4.4 × 10^−^^6^ M (*S*/*N* = 3), respectively, which is comparable with the reported results [12]. These results demonstrated that N-CDs/β-CD nanocomposites modified GCE can be used in the chiral detection of Trp enantiomers.

To confirm the feasibility, this electrochemical sensing platform was applied to detect the percentage composition of Trp enantiomer (l-Trp%) in racemic solution. Different racemic solutions were prepared by changing the proportion of l-Trp from 0 to 100% with the total concentration of 5.0 × 10^−3^ M. Figure 8D shows good linear relationships of *I*_p_ and ∆*E*_p_ (*E*_pD_ − *E*_p(D+L)_) with the percentage of l-Trp, which certifies that this method is able to determine Trp enantiomeric composition. Moreover, the regression equation between *I*_p_ and l-Trp% is expressed as *I*_p_ = −0.04248 + 8.7848 × 10^−5^ × l-Trp% with a correlation coefficient of 0.9924. The regression equation between ∆*E*_p_ and l-Trp% is expressed as ∆*E*_p_ = −0.9428 + 0.2148 × l-Trp% with a correlation coefficient of 0.9757. Such good chiral recognition ability is comparable to the best results reported previously [12,20].

The feasibility of this N-CDs/β-CD/GCE was further investigated by Trp enantiomers’ recognition in real samples. The riboflavin sample did not show any response of Trp enantiomers, indicating that the concentrations of Trp enantiomers in riboflavin sample were extremely low. Thus, 1.0 × 10^−^^3^ M l-Trp and d-Trp were added into the sample for recovery evaluation, and the recoveries ranged from 93.6% to 97.2% (*n* = 3) for l-Trp and 92.2% to 93.8% (*n* = 3) for d-Trp, addressing the good accuracy of this N-CDs/β-CD/GCE for Trp enantiomers’ recognition in real samples.

## 4. Conclusions

In the present work, an N-CDs/β-CD/GCE was easily constructed by efficiently electrodepositing N-CDs/β-CD nanocomposites on the GCE surface. The N-CDs/β-CD nanocomposites were characterized by spectroscopy methods, and the interaction mechanism between N-CDs/β-CD nanocomposites and Trp enantiomers was further investigated. The obtained results illustrated that the electrochemical oxidation of Trp enantiomers on N-CDs/β-CD/GCE was a two-electron two-proton transfer process, and Trp enantiomers bonded with N-CDs/β-CD to form single inclusion complex with molar ratio of one to one. Since l-Trp tended to preferentially bind to the N-CDs/β-CD nanocomposites, this N-CDs/β-CD/GCE, which combined the advantages of N-CDs and β-CD, allowed for the fabrication of a novel electrochemical sensing platform for enantioselective recognition of Trp enantiomers. Such a simple strategy may help to understand the selectivity of chiral compounds in biosystems and open up new opportunities for recognizing other chiral enantiomers in pharmaceutical assays and clinical industries.

## Supplementary Materials

The following are available online at http://www.mdpi.com/1424-8220/16/11/1874/s1, Figure S1: Influence of pH on the fluorescence intensity of N-CDs, Figure S2: DPVs responses of β-CD/GCE for l-Trp or d-Trp.
