# Supplementary material for: Novel N-Doped Carbon Dots/β-Cyclodextrin Nanocomposites for Enantioselective Recognition of Tryptophan Enantiomers
Source: Sensors (Basel). 2016 Nov 9;16(11):1874. doi: 10.3390/s16111874 (PMC5134533; doi:10.3390/s16111874)
Supplement: Supplementary file 1 [file sensors-16-01874-s001.pdf]

Sensors **2016**, *16*, 1874; doi:10.3390/s16111874

Supplementary Materials: Novel N-Doped Carbon Dots/β-Cyclodextrin Nanocomposites for Enantioselective Recognition of Tryptophan Enantiomers

Qi Xiao, Shuangyan Lu, Chusheng Huang, Wei Su and Shan Huang

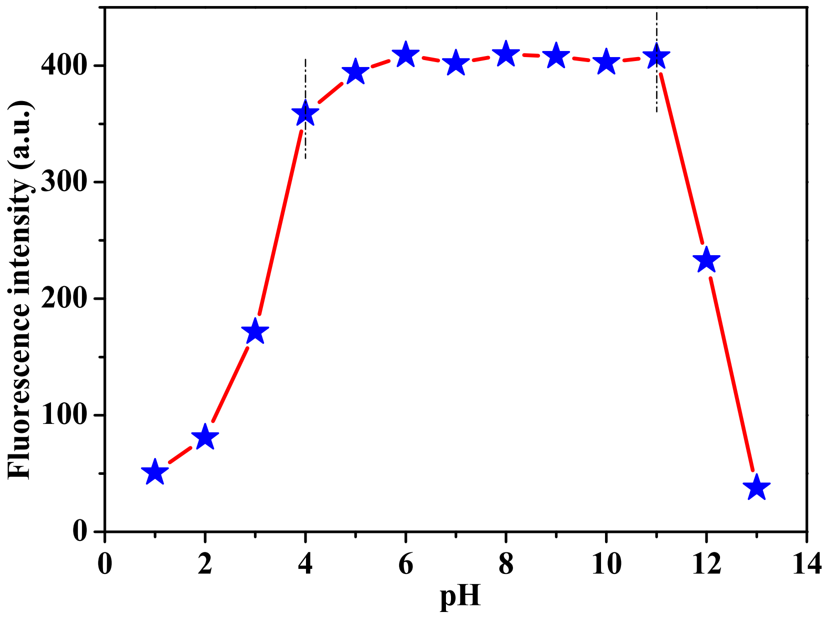

Figure S1. Influence of pH on the fluorescence intensity of N-CDs.

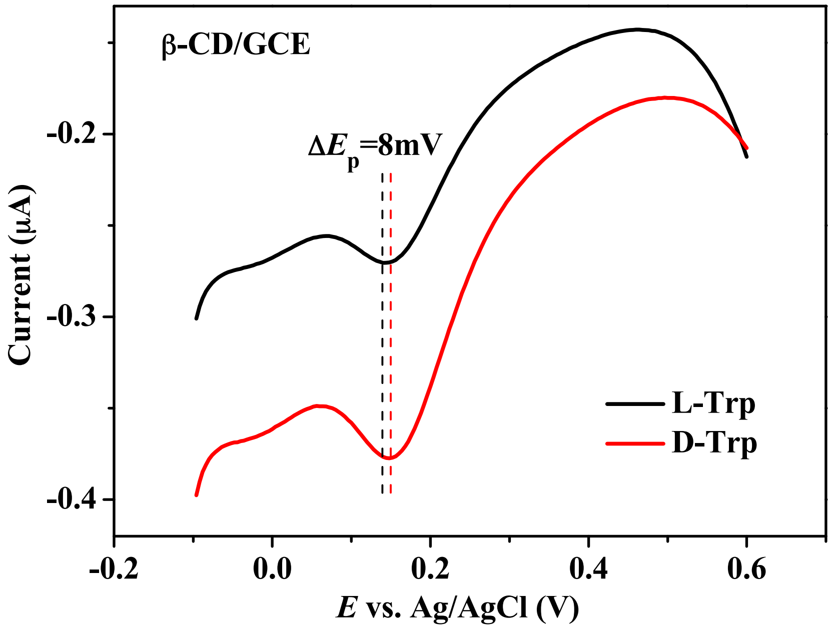

Figure S2. DPVs responses of β-CD/GCE for L-Trp or D-Trp.
